# C-terminal proendothelin-1 (CT-proET-1) is associated with organ failure and predicts mortality in critically ill patients

**DOI:** 10.1186/s40560-017-0219-y

**Published:** 2017-03-20

**Authors:** Lukas Buendgens, Eray Yagmur, Jan Bruensing, Ulf Herbers, Christer Baeck, Christian Trautwein, Alexander Koch, Frank Tacke

**Affiliations:** 1 Department of Medicine III, RWTH-University Hospital Aachen, Pauwelsstrasse 30, 52074 Aachen, Germany; 2 Medical Care Center, Dr. Stein and Colleagues, 41061 Mönchengladbach, Germany

**Keywords:** C-terminal proendothelin-1, CT-proET-1, ICU, Prognosis, Sepsis, Biomarker, Critical illness, Endothelin, ET-1

## Abstract

**Background:**

Endothelin 1 (ET-1) is a strong vasoconstrictor, which is involved in inflammation and reduced tissue perfusion. C-terminal proendothelin-1 (CT-proET-1) is the stable circulating precursor protein of ET-1. We hypothesized that CT-proET-1, reflecting ET-1 activation, is involved in the pathogenesis of critical illness and associated with its prognosis.

**Methods:**

Two hundred seventeen critically ill patients (144 with sepsis, 73 without sepsis) were included prospectively upon admission to the medical intensive care unit (ICU), in comparison to 65 healthy controls. CT-proET-1 serum concentrations were correlated with clinical data and extensive laboratory parameters. Overall survival was followed for up to 3 years.

**Results:**

CT-proET-1 serum levels at admission were significantly increased in critically ill patients compared to controls. CT-proET-1 serum levels showed significant correlations to systemic inflammation as well as multiple markers of organ dysfunction (kidney, liver, heart). Patients with sepsis displayed higher circulating CT-proET-1 than ICU patients with non-septic diseases. CT-proET-1 levels >74 pmol/L at ICU admission independently predicted ICU death (adjusted hazard ratio (HR) 2.66, 95% confidence interval (CI) 1.30–5.47) and overall mortality during follow-up (adjusted HR 2.19, 95%-CI 1.21–3.98).

**Conclusions:**

CT-proET-1 serum concentrations at admission are increased in critically ill patients and associated with sepsis, disease severity, organ failure, and mortality.

## Background

Endothelial dysfunction plays an important role in critical illness, especially in sepsis. It mediates hemodynamic disturbances based on the vasotonus, contributes to the balance of pro- and anti-inflammation, regulates nutrient supply and cell migration into tissue, and plays a key role in host-pathogen interaction [1]. Besides other mediators such as nitric oxide (NO), endothelin-1 (ET-1) is one of the major endogenous factors controlling vasotonus that is released from activated endothelial cells. It is the most prominent member of the endothelin family. It binds to two G-protein-coupled receptors, ET_A_ and ET_B_. ET_A_ promotes potent vasoconstriction and cell growth, whereas ET_B_ leads to vasodilation and inhibits cell proliferation [2]. Besides in blood vessels, ET-1-receptors are also found in tissues, e.g., cardiomyocytes and glomerular capillaries [3]. Endothelin release from endothelial cells is known to be stimulated by bacterial endotoxin [4] and various inflammatory cytokines such as TNF-alpha [3] or interleukin-6 [5] as well as mechanical factors like reduced shear stress [6].

Consecutively, increased levels of endothelin were found both in animal models of sepsis [7, 8] and human patients with sepsis [9–11]. Moreover, the function of many organs (e.g., liver, lung, heart, or kidney) worsens severely after infusion of ET-1 in animal models [12, 13]. In the past, various smaller studies could relate these findings to the clinical outcome of patients and demonstrated a relation between ET-1 and mortality in sepsis or septic shock in adults [9, 14, 15] and children [16]. ET-1 itself, however, is difficult to measure due to its limited half-life. Consequently, sample sizes of trials investigating ET-1 tend to be relatively small. The precursor peptide C-terminal proendothelin-1 (CT-proET-1) is far more stable and allows a stoichiometric measurement of ET-1 [17]. This facilitates the analysis of larger group of patients as well as the practical use of this potential biomarker in clinical routine. We therefore investigated CT-proET-1 in a large cohort of 217 consecutively enrolled critically ill patients, including 144 subjects with sepsis, in order to identify associations between CT-proET-1 and organ dysfunction, disease severity as well as ICU, and survival during follow-up in critically ill patients.

## Methods

### Study design

Written informed consent was obtained from the patient, his or her spouse, or the appointed legal guardian. Patients who were expected to have a short (<3 days) intensive care treatment (e.g., due to post-interventional observation or intoxication) were excluded [18]. The long-term course of patients was assessed by directly contacting the patient, the patients’ relatives, or their primary care physician. We used the third international consensus definitions for sepsis and septic shock (sepsis-3) as a post hoc definition for sepsis patients, and all others were classified as non-sepsis patients [19]. For identifying and classifying patients with an acute respiratory distress syndrome (ARDS), we used the Berlin definition of ARDS [20].

Sixty-five healthy blood donors with normal values for blood counts, C-reactive protein, and liver enzymes served as controls. The study protocol was approved by the local ethics committee and conducted in accordance with the ethical standards laid down in the 1964 Declaration of Helsinki (ethics committee of the University Hospital Aachen, RWTH-University, Aachen, Germany, reference number EK 150/06). The current study was part of a larger assessment of biomarkers in critically ill patients, conducted between 2006 and 2014 at our center.

### CT-proET-1 measurements

Blood samples were collected directly upon admission of the patient to the ICU prior to therapeutic interventions at the ICU. After centrifugation at 4 °C for 10 min, serum aliquots of 1 mL were frozen immediately at −80 °C. CT-proET-1 serum concentrations were measured using a commercially available fluorescent immunoassay (BRAHMS GmbH/ThermoFischer Scientific, Henningsdorf, Germany) following the manufacturer’s protocol. The scientist performing laboratory measurements was fully blinded to any clinical or other laboratory data of the patients or controls.

### Statistical analysis

Data are displayed as median and range due to the skewed distribution of most of the parameters. Differences between two groups were assessed by Mann-Whitney *U* test or chi-squared test. Differences between multiple groups were assessed using the Kruskal-Wallis test. To illustrate differences between subgroups, box plot graphics were used displaying a summary of the median, quartiles, range, and extreme values of the given data. Their whiskers range from the minimum to the maximum value excluding outliers displayed as separate points. An outlier was defined as a value that is smaller than the lower quartile minus 1.5 times the interquartile range or larger than the upper quartile plus 1.5 times the interquartile range. A far-out value was defined as a value that is smaller than the lower quartile minus three times the interquartile range or larger than the upper quartile plus three times the interquartile range [21]. Correlations between variables were assessed with Spearman correlation tests. The Cox regression model was used for univariate and multivariate analysis of risk factors. Kaplan-Meier curves were used to illustrate differences in survival [22]. Differences between the groups regarding survival were assessed with the log-rank test. Receiver operating characteristic (ROC) curve analysis were used to evaluate the value of a predictive marker or a composite score. ROC curves were generated by plotting sensitivity against 1-specificity [23]. Differences between ROC curves were assessed using the method described by DeLong et al. [24]. Statistical analyses were performed with SPSS Version 23 (SPSS, Chicago, IL, USA) and MedCalc Version 16 (MedCalc Software, Ostend, Belgium).

## Results

### CT-proET-1 serum concentrations are increased in critically ill patients and associated with sepsis

**Fig. 1:**
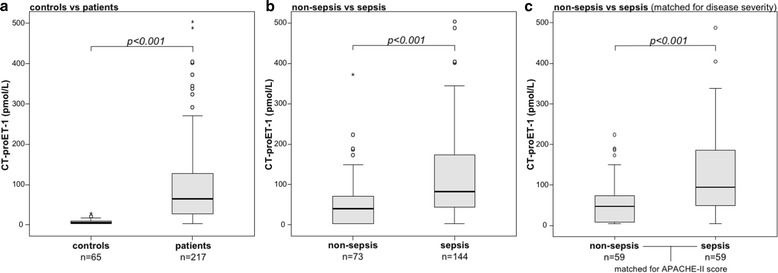
Serum CT-proET-1 concentrations in critically ill patients and sepsis. **a** Serum levels of CT-proET-1, at the time of admission to the ICU, were significantly higher in critically ill patients than in healthy controls (*p* < 0.001; *U* test). **b** CT-proET-1 levels were significantly higher in patients with sepsis (*p* < 0.001) compared to ICU patients without sepsis. **c** When matched 1:1 with non-sepsis patients for APACHE-II score, age, and sex, CT-proET-1 levels were significantly higher in patients with sepsis compared to ICU patients without sepsis (*p* < 0.001)

In order to investigate the role of CT-proET-1 in critical illness, we measured serum levels in 217 patients at the time of admission to our medical ICU. In comparison to 65 healthy controls, CT-proET-1 levels were strongly elevated in critically ill patients (median 5.8 vs 65.4 pmol/L, *p* < 0.001, *U* test; Fig. 1a).

Of all 217 patients, 144 were admitted because of sepsis. The most frequent septic focus was pneumonia (*n* = 74), followed by abdominal (*n* = 26) and urogenital infections (*n* = 11) (detailed data not shown). Non-septic ICU patients were admitted due to cardio-pulmonary diseases (*n* = 29), pancreatitis (*n* = 12), decompensated liver cirrhosis (*n* = 9), and other non-septic diseases (*n* = 23). CT-proET-1 levels were significantly higher in patients with sepsis compared to other ICU patients (median 40.9 vs 82.1 pmol/L; *p* < 0.001; Fig. 1b, Table 1). Sepsis and non-sepsis patients did not differ in age or sex, but sepsis patients had significantly higher APACHE-II or SOFA scores, increased mortality, longer stay on the ICU, and an increased vasopressor demand (Table 1). In order to exclude that the difference in CT-pro-ET1 was related to the presence of sepsis and not to disease severity, the non-sepsis ICU patients were matched 1:1 with a patient from the sepsis group for APACHE-II score (disease severity), age, and sex. In this subanalysis, CT-pro-ET1 remained indeed higher in the sepsis group (*p* < 0.001; Fig. 1c).

**Table 1:** Baseline patient characteristics and CT-proET-1 serum measurements at ICU admission

Parameter	All patients	Sepsis	Non-sepsis	*p* ^a^
Number	217	144	73	
Sex (male/female)	132/85	84/60	48/25	n.s.
Age median, (range) [years]	64 (18–90)	65 (20–90)	61 (18–85)	n.s.
APACHE-II score, median (range)	18 (2–43)	19 (4–43)	14 (2–33)	<0.001
SOFA score, median (range)	9 (0–17)	9 (2–17)	7 (0–17)	0.003
SAPS 2 score, median (range)	41 (0–73)	40 (0–73)	41 (0–73)	0.193
Mechanical ventilation, *n* (%)	144 (69)	98 (63)	46 (63)	n.s.
Ventilation time, median (range) [h]	117 (0–3828)	125.5 (0–2966)	66 (0–2828)	n.s.
Vasopressor demand, *n* (%)	132 (60.8)	99 (68.8)	33 (45.2)	<0.001
ICU days, median (range)	7 (1–137)	8.5 (1–137)	6 (1–45)	0.004
30-day mortality, *n* (%)	41 (18.9)	34 (23.6)	7 (9.6)	0.016
Overall mortality, *n* (%)	86 (41.7)	64 (46.7)	22 (31.9)	0.42
CT-proET-1, median (range) [pmol/L]	43.8 (3–503.6)	82.1 (3–503.6)	40.9 (3–372.9)	<0.001
Leucocytes, median (range) [per nL]	12.9 (0.5–208)	13.8 (0.5–208)	12.5 (1.8–29.6)	0.041
CRP, median (range) [mg/L]	98 (5–230)	160.5 (5–230)	17 (5–230)	<0.001
Cystatin C, median (range) [mg/L]	1.48 (0.39–8.38)	1.69 (0.39–8.38)	1.04 (0.56–2.29)	<0.001
Bilirubin, median (range) [per mg/dL]	0.7 (0.2–20.8)	0.7 (0.2–6.8)	0.7 (0.2–20.8)	n.s.

For quantitative variables, median and range (in parenthesis) are given

*Abbreviations*: *CRP* C-reactive protein, *CT-proET-1* C-terminal proendothelin-1, *APACHE* Acute Physiology and Chronic Health Evaluation, *SAPS 2* Simplified Acute Physiology Score, *SOFA* Sequential Organ Failure Assessment, *n.s.* not significant

^a^Significance between sepsis and non-sepsis patients was assessed using the Mann-Whitney *U* test or chi-squared test

To investigate a possible use of CT-proET-1 in identifying patients with sepsis, we conducted a ROC curve analysis comparing CT-proET-1 to other, established markers (procalcitonin and C-reactive protein (CRP)). With an AUC of 0.834 (95%-CI 0.768–0.900), CRP was significantly superior to both PCT (*p* = 0.046; DeLong test) and CT-proET-1 (*p* = 0.007; DeLong test). Interestingly, CT-proET-1 was non-inferior to PCT (AUC 0.704 vs 0.757; *p* = 0.24; DeLong test).

### CT-proET-1 levels in critically ill patients correlate with clinical disease severity scores and organ dysfunction

Based on the pathogenic role of endothelin-1 for vasoconstriction and impaired tissue perfusion [25], we hypothesized that increased CT-proET-1 might be associated with organ dysfunction in ICU patients. Strikingly, CT-proET-1 levels were strongly associated with markers of renal dysfunction (e.g., creatinine, *r* = 0.500, *p* < 0.001; cystatin C, *r* = 0.624, *p* < 0.001), cholestasis (e.g., bilirubin, *r* = 0.148, *p* = 0.031), impaired hepatic synthesis (e.g., albumin, *r* = −0.321, *p* = 0.001; pseudocholinesterase, *r* = −0.438, *p* < 0.001; prothrombin time, *r* = −0.220, *p* = 0.001) and cardiac failure (e.g., brain natriuretic peptide, *r* = 0.505, *p* < 0.001). Likewise, CT-proET-1 levels correlated with markers of general inflammation (e.g., C-reactive protein, *r* = 0.416, *p* < 0.001, Table 2).

**Table 2:** Correlations of CT-proET-1 with clinical scores and other laboratory markers

	All patients	
	*r*	*p*
Markers of inflammation		
CRP	0.416	<0.001
Procalcitonin	0.343	0.005
IL6	0.172	0.027
Markers of organ dysfunction		
Cystatin C	0.624	<0.001
GFR	−0.534	<0.001
ALT	−0.165	0.016
Bilirubin	0.148	0.031
Prothrombin time	−0.220	0.001
Albumin	−0.321	<0.001
Urea	0.577	<0.001
NTproBNP	0.505	<0.001
Clinical scores		
APACHE-II	0.239	0.001
SOFA	0.136	n.s.
SAPS 2	0.400	<0.001
New and experimental biomarkers		
Resistin	0.449	<0.001
NTproCNP	0.604	<0.001
suPAR	0.529	<0.001

*Abbreviations*: *ALT* alanine aminotransferase, *APACHE* Acute Physiology and Chronic Health Evaluation, *ALT* alanine aminotransferase, *CRP* C-reactive protein, *GFR* glomerular filtration rate, *IL6* interleukin 6, *NTproBNP* amino-terminal propeptide of brain natriuretic peptide, *NTproCNP*, amino-terminal propeptide of C-type natriuretic peptide, *SAPS* Simplified Acute Physiology Score, *SOFA* sequential organ failure assessment, *suPAR* soluble urokinase plasminogen activator receptor, *n.s.* not significant

**Fig. 2:**
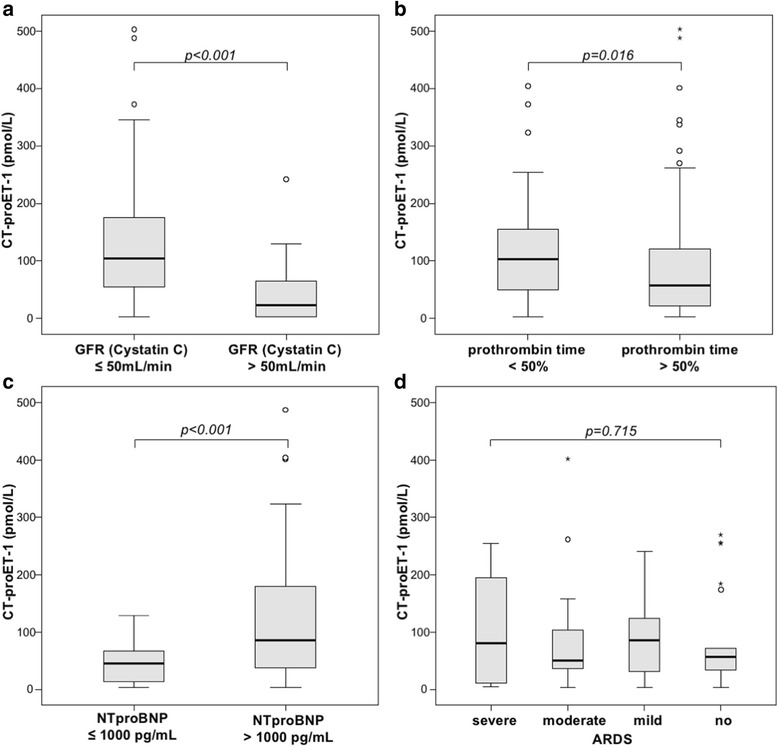
Serum CT-proET-1 levels in critically ill patients are associated with organ failure. **a**–**c** Serum levels of CT-proET-1 at the time of admission to the ICU were significantly higher in critically ill patients with renal (cystatin C-based glomerular filtration rate (GFR) <50 mL/min; *p* < 0.001, *U* test), hepatic (prothrombin time <50%; *p* = 0.016) or cardiac failure (NTproBNP >1000 pg/mL; *p* < 0.001). **d** CT-proET-1 was not associated with the presence or severity of an acute respiratory distress syndrome (*p* = 0.715, Kruskal-Wallis test)

Interestingly, patients with manifest organ failure had significantly elevated CT-proET-1 levels. This was observed for patients with renal failure (defined as a cystatin C-based glomerular filtration rate below 50 mL/min, Fig. 2a), liver failure (defined as prothrombin time <50%, Fig. 2b), or heart failure (defined as a NTproBNP >1000 pg/ml; Fig. 2c). As prior studies reported elevated ET-1 in patients with ARDS compared to controls [26], we further assessed CT-proET-1 serum levels in regard to the degree of an ARDS. However, CT-proET-1 did not differ between ICU patients without ARDS (*n* = 22) and with mild (*n* = 29), moderate (*n* = 27), or severe (*n* = 13) ARDS at the time of admission (Fig. 2d).

**Fig. 3:**
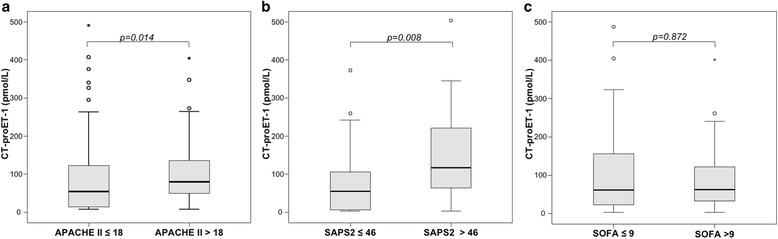
Serum CT-proET-1 levels in critically ill patients are associated with disease severity. Critically ill patients with higher disease severity as represented by APACHE-II (**a**), SAPS 2 (**b**) showed significantly higher CT-proET-1 levels, while SOFA score (**c**) was unrelated to CT-proET-1

Additionally, CT-proET-1 levels were associated with severity of critical illness. Patients with higher APACHE-II (above 18) and SAPS 2 (above the median of the cohort) scores showed significantly increased serum levels of CT-proET-1 (Fig. 3a, b). CT-proET-1 also positively correlated with these disease severity scores (APACHE-II, *r* = 0.239, *p* = 0.001; SAPS 2, *r* = 0.400, *p* < 0.001, Table 2). CT-proET1 did not correlate with the SOFA score, neither for all nor for sepsis patients (detailed data not shown).

### CT-proET-1 at admission is an independent predictor of ICU mortality

**Fig. 4:**
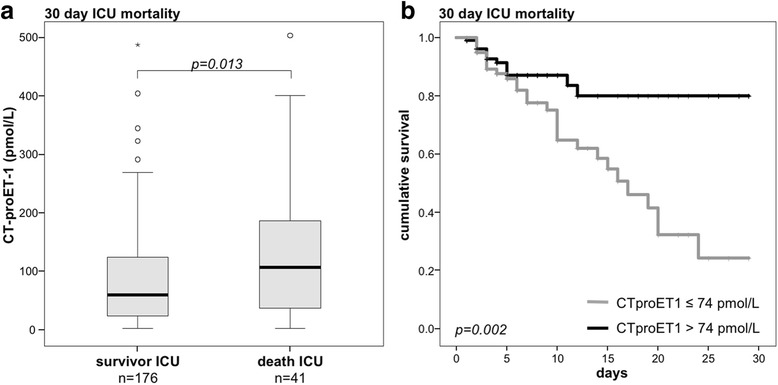
Prediction of ICU mortality by CT-proET-1 serum levels. **a** Patients that died during the course of ICU treatment had significantly higher serum CT-proET-1 levels on admittance to ICU (*p* < 0.001; *U* test) than survivors. **b** Kaplan-Meier survival curves of ICU patients are displayed for the 30-day mortality, showing that patients with CT-proET-1 below a cut-off value of 74 pmol/L had a better outcome at the ICU (*p* = 0.002; log-rank test)

As CT-proET-1 levels correlate with organ dysfunction and disease severity, we hypothesized that CT-proET-1 serum concentrations at the time of ICU admission might be associated with mortality in critically ill patients. Overall, *n* = 41 (18.9%) of the patients died at the ICU, while *n* = 86 (39.6%) died overall including the follow-up time (of up to 3 years). Remarkably, patients that died at the ICU showed significant higher serum levels of CT-proET-1 at ICU admission than survivors (median 88.3 vs 59.2 pmol/L; *p* = 0.029; Fig. 4a).

By Cox regression analysis, CT-proET-1 levels were found to predict ICU mortality (*p* = 0.047). We used the Youden index (28) to find the best cut-off value regarding sensitivity and specificity. Based on the coordinates of the ROC curve, a CT-proET-1 cut-off value of 74 pmol/L showed the best ratio of sensitivity and specificity in predicting ICU mortality. Interestingly, this value is higher than the measurements of our healthy controls and represents the 99th percentile of a healthy population. Kaplan-Meier survival curve analysis confirmed that high CT-proET-1 levels were strongly associated with 30-day mortality (Fig. 4b; *p* = 0.002). As CT-proET-1 correlates with markers of organ failure, excretion and inflammation, we next tested if CT-proET-1 serum levels can independently predict survival. We performed uni- and multivariate Cox regression analysis including age, markers of inflammation (i.e., CRP), renal (i.e., creatinine), circulatory (i.e., lactate), and hepatic dysfunction (i.e., bilirubin, prothrombin time). Here, high CT-proET-1 (>74 pmol/L) was an independent predictor of ICU mortality in critically ill patients (adjusted hazard ratio (HR) 2.66 (95% CI 1.30–5.47), Table 3).

**Table 3:** Uni- and multivariate Cox regression analyses for CT-proET-1 levels at ICU admission to predict ICU mortality

	Unadjusted HR (95%-CI)	*p*	Adjusted HR (95%-CI)	*p*
CT-proET-1 > 74 pmol/L	2.658 (1.375–5.137)	0.004	2.663 (1.296–5.470)	0.008
Creatinine (per mg/dL)	–	n.s.		
CRP (per mg/L)	–	n.s.		
Bilirubin (per mg/dL)	1.129 (1.021–1.249)	0.018	–	n.s.
Prothrombin time (per %)	0.988 (0.977–1)	0.045	–	n.s.
Lactate (per mmol/L)	1.115 (1.03–1.207)	0.007	1.20 (1.109–1.298)	<0.001
Age (per year)	1.034 (1.011–1.058)	0.003	1.045 (1.019–1.71)	0.003

Variables with an univariate *p* < 0.25 were included in the multivariate model

*Abbreviations*: *95%-CI*, 95% confidence interval, *CRP* C-reactive protein, *CT-proET-1* C-terminal proendothelin-1, *n.s.* not significant

### High levels of CT-proET-1 at ICU admission are associated with overall survival

**Fig. 5:**
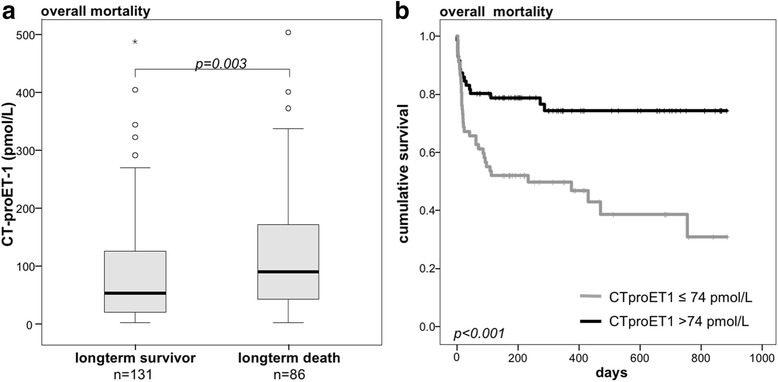
Prediction of overall mortality by CT-proET-1 serum concentrations. **a** Patients that died during the total observation period had significantly higher serum CT-proET-1 levels at ICU admission than survivors (*p* < 0.001; *U* test). **b** Kaplan-Meier survival curves of ICU patients are displayed, showing that patients with CT-proET-1 levels above a cut-off of 74 pmol/L have an increased overall mortality (*p* < 0.001; log-rank test)

Given its association with short-term mortality, we examined if the level of CT-proET-1 at admission to the ICU was also related to long-term outcome. We found that CT-proET-1 levels were significantly higher in patients that died during the follow-up period compared to the overall surviving patients (median 53.7 vs 89.9 pmol/L; *p* = 0.003; Fig. 5a).

Again, Kaplan-Meier analysis showed a good discrimination in terms of long-term survival between the groups with high and low CT-proET-1 values (Fig. 5b, *p* < 0.001).

In addition, we conducted uni- and multivariate Cox regression analyses including age, markers of inflammation (i.e., CRP) and renal (i.e., creatinine), circulatory (i.e., lactate), and hepatic dysfunction (i.e., bilirubin, prothrombin time) (Table 4). Adjusted by the factors above high CT-proET-1 (>74 pmol/L) remained an independent predictor of overall survival (adjusted HR 2.19, 95%-CI 1.21–3.98).

**Table 4:** Uni- and multivariate Cox regression analyses for CT-proET-1 levels at ICU admission to predict overall mortality

	Unadjusted HR (95%-CI)	*p*	Adjusted HR (95%-CI)	*p*
CT-proET-1 > 74 pmol/L	2.731 (1.535–4.858)	0.001	2.193 (1.209–3.975)	0.01
Creatinine (per mg/dL)	1.034 (0.946–1.129)	0.461	–	n.s.
CRP (per mg/L)	1.002 (0.999–1.006)	0.129	–	n.s.
Bilirubin (per mg/dL)	1.16 (0.935–1.16)	0.176	–	n.s.
Prothrombin time (per %)	0.99 (0.979–1)	0.052	–	n.s.
Lactate (per mmol/L)	1.082 (0.949–1.234)	0.239	–	n.s.
Age (per year)	1.036 (1.014–1.059)	<0.001	1.028 (1.005–1.051)	0.04

Variables with a univariate *p* < 0.25 were included in the multivariate model

*Abbreviations*: *95%-CI* 95% confidence-interval, *CRP* C-reactive protein, *CT-proET-1* C-terminal proendothelin-1, *n.s.* not significant

## Discussion

In this study, we demonstrate striking regulations of circulating CT-proET-1 in a large cohort of critically ill patients, supporting our hypothesis to use this stoichiometric indicator of ET-1 as a diagnostic and prognostic biomarker in intensive care medicine. We could show that CT-proET-1 is significantly elevated in critically ill patients compared to healthy controls, correlates with disease severity and organ failure, and is an independent risk predictor for ICU and overall mortality.

Previous smaller studies [9, 14, 15] had reported an association of CT-proET-1 with short-term mortality in patients with sepsis. In contrast, one study on 99 sepsis patients that measured CT-proET-1 within 48 h after admission did not reproduce the relation between CT-proET-1 and mortality risk [27]. Very recently, Lundberg and colleagues reported that elevated CT-proET-1 levels at ICU admission were associated with 7-day and 28-day mortality in 53 patients with septic shock [14]. Our study now extends these prior findings to a more heterogeneous, larger prospective cohort of medical ICU patients demonstrating a clear prognostic value of circulating CT-proET-1. While patients with sepsis had higher CT-proET-1 levels than ICU patients with non-septic disease, CT-proET-1 at ICU admission predicted 30-day mortality for the total patient cohort as well as for sepsis or non-sepsis patients. Moreover, we found that CT-proET-1 levels at ICU admission were even indicative of the long-term mortality risk, based on follow-up observations of about 3 years. This effect of CT-proET-1 on ICU and overall mortality was independent from single markers of organ failure or inflammation, indicating that CT-proET-1 could be useful in clinical algorithms or scores aiming at identifying high-risk patients upon ICU admission.

A prior study by Druml et al. found an association between ET-1 and the presence of an ARDS [26]. We did not observe such an association, but our study population was considerably different. While our cohort included a rather heterogeneous population of medical ICU patients with and without sepsis, Druml et al. specifically investigated ARDS patients in comparison to healthy controls, presumably with fewer confounding comorbidities than in our patient population.

Moreover, our findings might also provide important insights into the role of ET-1 (measured by CT-proET-1) in the pathogenesis of critical illness. The serum levels of CT-proET1 correlated with organ dysfunction and disease severity, but not with lactate, a marker of shock and circulatory failure. Thus, CT-proET1 reflects not a just mere hypoperfusion of tissues due to circulatory failure, but a more complex endothelial activation or dysfunction related to organ failure.

Nonetheless, our exploratory study has several limitations. These include the single center setting with the retrospective assessment of CT-proET1 in a prospectively enrolled study cohort. Moreover, we do not have longitudinal measurements of CT-proET1, which could potentially improve the prognostic validity of this marker. Also, organ failure assessment was solely based on laboratory parameters, but no functional tests (like echocardiography or liver biopsy).

However, the hyperactivation of ET-1 in our cohort of ICU patients and the strong association of ET-1 with organ dysfunction and mortality indicate that ET-1 might be a potential drug target in critical illness and sepsis. It is tempting to speculate that antagonizing systemic supra-physiological ET-1 levels holds therapeutic potential to improve tissue perfusion. In fact, the endothelin receptor antagonist bosentan has shown positive effects on tissue perfusion [28, 29] and cardiac output [30, 31] in animal models of septic shock. Moreover, the application of bosentan in a rodent model of septic shock was even able to improve survival [32]. Interestingly, this effect was more pronounced, if bosentan was given in the hypodynamic stage after fluid resuscitation, a disease stage where specific treatment options are currently scarce. Our data corroborate to further investigate ET-1-antagonistic approaches in the ICU setting, in order to define the efficacy as well as optimal dose and timing for such an intervention.

## Conclusions

Our study shows that CT-proET-1 is elevated in critically ill patients and in sepsis. It is associated with organ dysfunction and poses an independent risk factor for ICU and overall mortality. The potential as a drug treatment target in critically ill patients requires further investigations.

## Abbreviations

95%-CI: 95% confidence interval
APACHE: Acute Physiology and Chronic Health Evaluation
ARDS: Acute respiratory distress syndrome
AUC: Area under the curve
CRP: C-reactive protein
CT-proET-1: C-terminal proendothelin-1
ET-1: Endothelin-1
HR: Hazard ratio
ICU: Intensive care unit
NTproBNP: N-terminal B-type natriuretic peptide
ROC: Receiver operating characteristics
SAPS 2: Simplified acute physiology score
SOFA: Sequential Organ Failure Assessment
